# Diversification and prevalence of the quinolone resistance *crpP* genes and the *crpP*-carrying Tn*6786*-related integrative and conjugative elements in *Pseudomonas aeruginosa*

**DOI:** 10.1080/21505594.2021.1962160

**Published:** 2021-08-17

**Authors:** Zhichen Zhu, Huiying Yang, Zhe Yin, Ying Jing, Yuee Zhao, Hongyu Fu, Hong Du, Dongsheng Zhou

**Affiliations:** a Department of Clinical Laboratory, The Second Affiliated Hospital of Soochow University, Suzhou, China; b State Key Laboratory of Pathogen and Biosecurity, Beijing Institute of Microbiology and Epidemiology, Beijing, China

**Keywords:** *Pseudomonas aeruginosa*, quinolone resistance, *crpP*, integrative and conjugative element, typing/nomenclature scheme, China

## Abstract

The quinolone resistance *crpP* genes can mediate decreased susceptibility to quinolones. However, diversification and prevalence of *crpP* genes and *crpP*-carrying integrative and conjugative elements (ICEs) still need to be elucidated. In this study, genome sequencing was conducted for 200 Chinese *Pseudomonas aeruginosa* isolates, 16 of which were fully sequenced. All the 37 available CrpP variants were collected for phylogenetic analysis, 10 CrpP enzymes were chosen to conduct cloning and antimicrobial susceptibility test, and 22 *crpP*-carrying Tn*6786*-related ICEs were selected for detail genetic dissection analysis. Then, typing/nomenclature schemes for *crpP* variants and *crpP*-carrying ICEs were established for the first time. The 10 representative CrpP enzymes were confirmed to mediate decreased susceptibility to one to three quinolones. Tn*6786*-related ICEs displayed high-level diversification in both nucleotide sequences and modular structures. Mainly, massive gene acquisition/loss occurred across the whole genomes of Tn*6786*-related ICEs. 53.5% (107/200) of the tested clinical *P. aeruginosa* isolates from China carried *crpP* genes, which were exclusively located within chromosome-borne Tn*6786*-related ICEs. The *crpP*-carrying ICEs were at active stages of evolution and had the high potential to be an important vector for the dissemination of resistance genes besides *crpP*. The present study furthered the understanding of the bioinformatics and epidemiology of *crpP* genes and *crpP*-carrying ICEs.

## Introduction

There are at least three major mechanisms of resistance to quinolones in bacteria: mutations in genes encoding quinolone-targeting proteins; changes in expression of efflux pumps or porin channels; and acquisition of quinolone resistance genes such as *crpP* [1]. The CrpP enzyme can induce ATP-dependent phosphorylation of ciprofloxacin and mediate a decreased susceptibility to ciprofloxacin [2,3]. Abundant *crpP* homologues have been mainly identified in *Pseudomonas aeruginosa* [4–9], and less frequently in *Acinetobacter baumannii* [4], *Escherichia coli*, and *Klebsiella pneumoniae* [10]; however, a typing/nomenclature scheme for *crpP* variants is still lacking.

Integrative and conjugative elements (ICEs) [11–13] are important mobile genetic elements (MGEs) involved in the dissemination of resistance genes and other beneficial genes in bacteria. An ICE element can transfer between cells because of their self-encoded conjugation function, and it is typically composed of *attL* (attachment site at the left end), *int* (integrase), *xis* (excisionase), *rlx* (relaxase), *oriT* (origin of conjugative replication), *cpl* (coupling protein), a P (TivB)- or F (TivF)-type T4SS gene set (mating pair formation), and *attR* (attachment site at the right end). While ICEs have been identified as a major *crpP* reservoir in *P. aeruginosa* [14], there are still no reports on systematic classification and detailed genetic dissection of *crpP*-carrying ICEs.

This study presented the systematic typing/nomenclature schemes for *crpP* variants and *crpP*-carrying ICEs, and confirmed a limited decreased susceptibility to quinolones mediated by 10 major representative CrpP enzymes. 53.5% (107/200) of the clinical *P. aeruginosa* isolates tested from China carried the *crpP* genes, which were exclusively located within chromosome-borne Tn*6786*-related ICEs. A very detailed genetic dissection analysis of 22 representative *crpP*-carrying Tn*6786*-related ICEs revealed that they displayed high-level diversification in both nucleotide sequences and modular structures. Especially, massive gene acquisition/loss occurred across the whole genomes of Tn*6786*-related ICEs. The reported data provided a deeper insight into the bioinformatics and epidemiology of *crpP* genes and *crpP*-carrying Tn*6786*-related ICEs.

## Materials and methods

A total of 200 *P. aeruginosa* isolates (Supplementary material Table S1) causing nosocomial infections were collected from 18 Chinese hospitals between 2010 and 2019. Draft-genome sequencing of all these 200 isolates was conducted using a paired-end library with an average insert size of 350 bp (ranged from 150 bp to 600 bp) on a HiSeq sequencer (Illumina, CA, USA). In addition, 16 of these 200 isolates were subjected to complete-genome sequencing, which was performed with a sheared DNA library with average size of 15 kb (ranged from 10 kb to 20 kb) on a PacBio RSII sequencer (Pacific Biosciences, CA, USA) (Supplementary material Table S1), and further sequence data mining was performed as previously described [15,16]. Bacterial sequence types (STs) were identified according to the online *P. aeruginosa* MLST scheme (https://pubmlst.org/paeruginosa). Indicated amino acid or nucleotide sequences were aligned using *Clustal Omega* 1.2.2 [17]. Unrooted maximum-likelihood phylogenetic trees were generated using *MEGAX* 10.1.8 [18] with a bootstrap iteration of 1000. *GraphPad Prism* 5.0 statistical software was used for statistical analysis. The χ2 was used for categorical variables. *P* < 0.05 was considered statistically significant. Additionally, different Tn numbers were used to name ICEs as previously described [19,20].

Conjugal transfer of each indicated ICE from its wild-type isolate into the rifampin-resistant *P. aeruginosa* ATCC 15,692 was conducted as previously described [16]. The *crpP-1.1* coding region together with its immediately 288-bp upstream promoter-proximal region and 216-bp downstream terminator-proximal region from the wild-type isolate SE5416 was cloned into the *lacZ* gene of kanamycin-resistant cloning vector pUC57K. Similarly, the coding region of each of the other indicated *crpP* variants together with the above promoter- and terminator-proximal regions were synthesized and cloned into pUC57K. Each resulting recombinant plasmid was transformed through electroporation into *Escherichia coli* TOP10 to generate the relevant electroporant. Bacterial antimicrobial susceptibility was tested by the classic broth microdilution method, and was interpreted as per the 2020 Clinical and Laboratory Standards Institute (CLSI) guidelines [21].

The complete chromosome sequences of the 201,330, A0002, SE5369, SE5416, SE5452, SE5431, SE5381, SE5429, SE5418, SE5357, T12746, YTSY4, 2,011,305, SE5430, SE5443, and SE5458 isolates were submitted to GenBank under accession numbers CP054623, CP054794, CP046403, CP046404, CP054793, CP054792, CP054789, CP054845, CP054790, CP054844, CP045552, CP054788, CP054787, CP054791, CP046405, and CP046406, respectively. The remaining 186 genome sequences were submitted to GenBank under BioProject PRJNA671779.

## Results

### A typing/nomenclature scheme for CrpP variants

**Figure 1.**
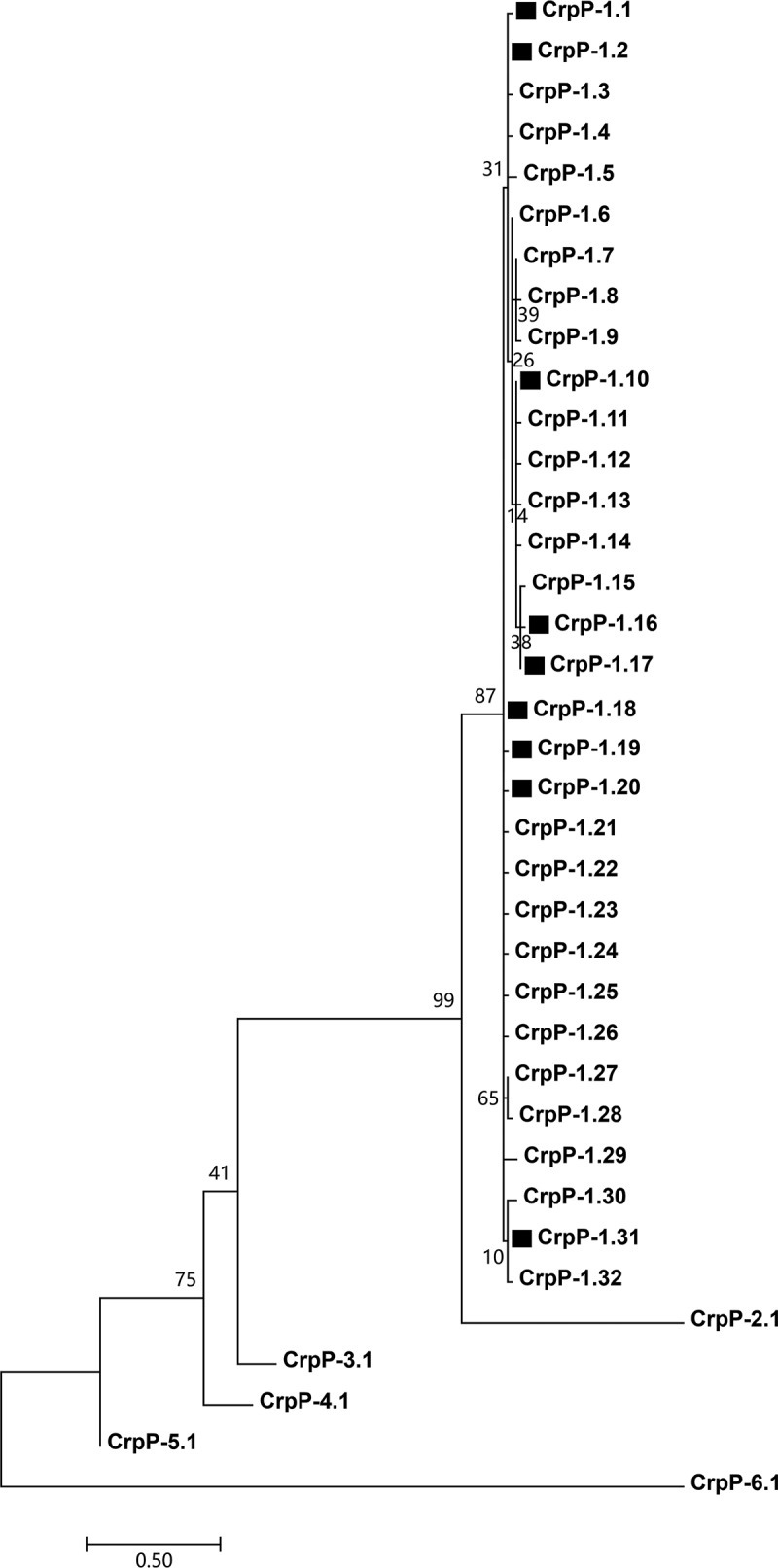
Evolution relationships of the 37 CrpP variants. Squares denote the nine CrpP variants detected in the 200 *P. aeruginosa* isolates sequenced in this study. Degree of support (percentage) for each cluster of associated taxa, as determined by bootstrap analysis, is shown next to each branch. Bar corresponds to scale of sequence divergence

A phylogenetic tree (Figure 1) was constructed from the aligned amino acid sequences of all the 37 available CrpP variants (Supplementary material Table S2), which were collected from previous reports [2,4–10]. These 37 CrpP variants were divided into six major separately clustering clades, which were designated as the CrpP primary groups CrpP-1 to CrpP-6. Based on amino acid substitution within CrpP sequences, the largest primary group CrpP-1 could be further divided into 32 variants CrpP-1.1 to CrpP-1.32, while each of the other five primary CrpP groups contained only a sole variant: CrpP-2.1, CrpP-3.1, CrpP-4.1, CrpP-5.1, and CrpP-6.1. Herein, the initially identified CrpP enzyme [2] was named as CrpP-1.1.

### CrpP-mediated limited decreased susceptibility to quinolones

The 10 representative *crpP* variants *crpP-1.1*/*1.10*/*1.16*/*1.20*/*1.31*/*2.1*/*3.1*/*4.1*/*5.1*/*6.1* were cloned into pUC57K, and the resulting recombinant plasmids were transformed into TOP10 to obtain the 10 corresponding *E. coli* electroporants pUC57K-*crpP-1.1*/*1.10*/*1.16*/*1.20*/*1.31*/*2.1*/*3.1*/*4.1*/*5.1*/*6.1*–TOP10, respectively. The subsequent antimicrobial susceptibility test involved a collection of 13 *E. coli* strains, including the above 10 *crpP*-carrying electroporants, the empty electroporant TOP10/pUC57K, the recipient strain TOP10, and the control strain ATCC 25,922 (Table 1). All these ten CrpP enzymes mediated a 2-fold to 4-fold decreased susceptibility to ciprofloxacin. Furthermore, CrpP-1.10/1.16/1.20/1.31 mediated a 2-fold to 8-fold decreased susceptibility to levofloxacin, and CrpP-1.31 mediated a 2-fold decreased susceptibility to norfloxacin.

**Table 1.** Antimicrobial drug susceptibility profile

Bacterial isolate	Minimum inhibitory concentration (mg/L)/antimicrobial susceptibility					
	Nalidixic acid	Norfloxacin	Ciprofloxacin	Levofloxacin	Moxifloxacin	Kanamycin
TOP10/pUC57K-*crpP-1.1*	2/S	0.03/S	0.008/S	0.008/S	0.03	≥64/R
TOP10/pUC57K-*crpP-1.10*	2/S	0.03/S	0.016/S	0.032/S	0.03	≥64/R
TOP10/pUC57K-*crpP-1.16*	2/S	0.03/S	0.016/S	0.064/S	0.03	≥64/R
TOP10/pUC57K-*crpP-1.20*	2/S	0.03/S	0.016/S	0.064/S	0.03	≥64/R
TOP10/pUC57K-*crpP-1.31*	2/S	0.06/S	0.016/S	0.016/S	0.03	≥64/R
TOP10/pUC57K-*crpP-2.1*	2/S	0.03/S	0.008/S	0.008/S	0.03	≥64/R
TOP10/pUC57K-*crpP-3.1*	2/S	0.03/S	0.016/S	0.008/S	0.03	≥64/R
TOP10/pUC57K-*crpP-4.1*	2/S	0.03/S	0.008/S	0.008/*S*	0.03	≥64/R
TOP10/pUC57K-*crpP-5.1*	2/S	0.03/S	0.008/S	0.008/S	0.03	≥64/R
TOP10/pUC57K-*crpP-6.1*	2/S	0.03/S	0.008/S	0.008/S	0.03	≥64/R
TOP10/pUC57K	2/S	0.03/S	0.004/S	0.008/S	0.03	≥64/R
TOP10	2/S	0.016/S	0.004/S	0.008/S	0.03	2/S
ATCC 25,922	2/S	0.06/S	0.016/S	0.03/S	0.06	4/S

S = sensitive; R = resistant.

### *A typing/nomenclature scheme for* crpP*-carrying Tn*6786*-related ICEs*

**Figure 2.**
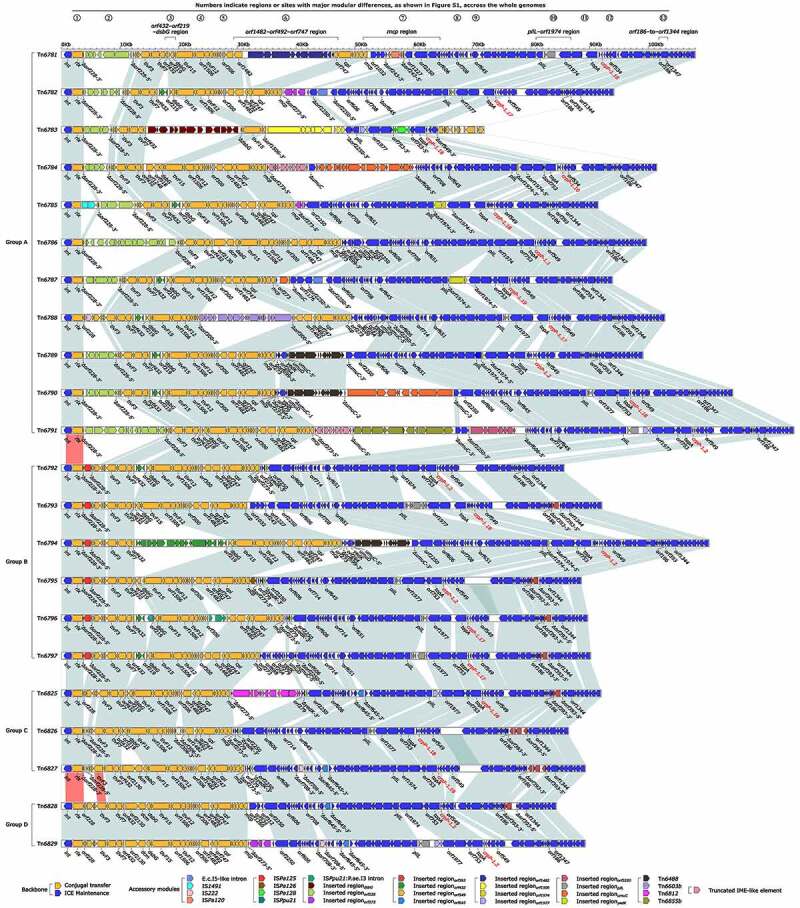
Linear comparison of the 22 *crpP*-carrying Tn*6786*-related ICEs. Genes are denoted by arrows. Genes, MGEs and other features are colored based on function classification. Shading regions denote homology of ICE backbone regions (light blue: ≥90% nucleotide identity; light red: 80% to 90% nucleotide identity)

**Figure 3.**
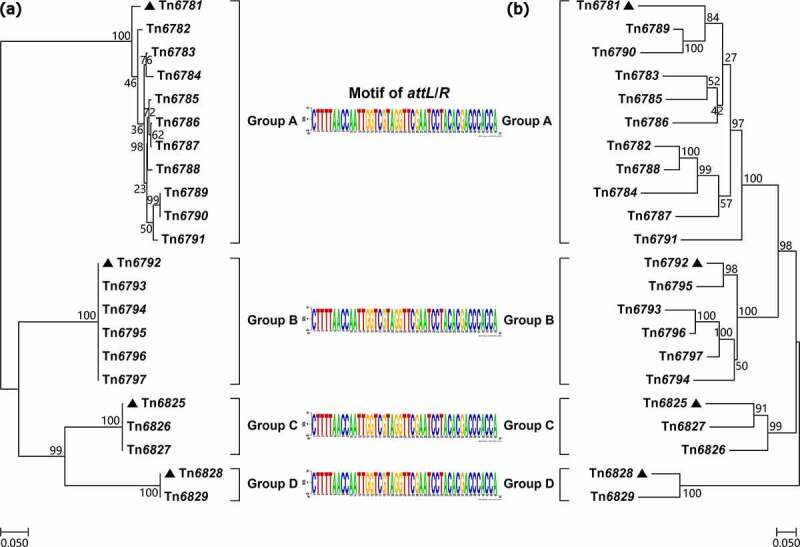
Evolution relationships of the 22 Tn*6786*-related ICEs. Two maximum likelihood phylogenetic trees are constructed from the aligned *int* sequences (a) and from the ICE core backbone SNPs (b), respectively. Triangles indicate the first sequenced ICEs of groups A to D, respectively. Degree of support (percentage) for each cluster of associated taxa, as determined by bootstrap analysis, is shown next to each branch. Bar corresponds to scale of sequence divergence

A detailed genetic dissection analysis was applied to a total of 22 representative fully sequenced *crpP*-carrying ICEs (Supplementary material Table S3). The modular structure of each ICE was divided into the backbone, and the accessory modules, which were defined as exogenous DNA regions, were inserted at different sites of the backbone (Figure 2). These 22 ICEs had similar backbone gene organizations, but they displayed >91% nucleotide identity with coverage from 28% to 98% of their backbone sequences (Supplementary material Table S4), thus indicating high-level diversification in their nucleotide sequences and modular structures. These 22 ICEs shared the core backbone markers *int, rlx*, an F-type T4SS gene set, *crpP-1*, and 45-bp *attL/R*; totally, six *crpP-1* variants were identified (Figure 2, and supplementary material Table S5). The phylogenetic tree constructed from the aligned *int* sequences (Supplementary material Table S6) and that from the core single-nucleotide polymorphisms (SNPs) within the ICE backbones indicated that these 22 ICEs could be divided into four separate clustering groups that were designated as groups A to D (Figure 3). As shown by pairwise comparison of *int* sequences, ICEs within each of these four groups shared >90% nucleotide identity, whereas <90% sequence identity was observed between different groups (Supplementary material Table S6).

### *Massive gene acquisition/loss in* crpP*-carrying Tn*6786*-related ICEs*

At least 13 regions/sites across the ICE genomes were recognized to display the major modular differences among these 22 ICEs (Supplementary material Figure S1 and Table S7). Firstly, a total of 97 events of exogenous gene acquisition occurred at these 13 regions/sites in these 22 ICEs, which carried different profiles of accessory modules (Supplementary material Figure S2-S8). Tn*6786* was identified as the reference of this ICE group because it carried a sole accessory module and possessed the mostly intact backbone (83.7 kb in length) among these 22 ICEs. Additionally, it harbored the firstly discovered *crpP* variant *crpP-1.1* (Figure 2). Each of the other 21 ICEs acquired two to seven accessory modules, which included IS elements, unit transposons, introns, and so called “inserted regions” [19]. Secondly, a total of 69 events of deletion of backbone regions, approximately a half of which resulted from exogenous gene acquisition, occurred at seven region/sites in 18 ICEs. Among each of these, seven experienced at least one event of deletions of >5-kb backbone regions. Tn*6783* had the shortest backbone that was 25.5 kb in length and 58.2-kb shorter than Tn*6786*. Finally, there were totally 10 events substitutions of backbone regions at two region/sites in nine ICEs.

### *Prevalence of* crpP *genes and* crpP*-carrying ICEs in Chinese* P. aeruginosa *isolates*

**Figure 4.**
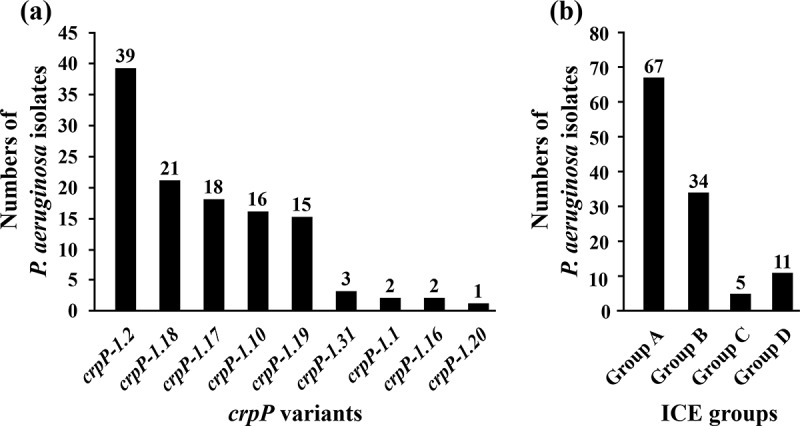
Prevalence of *crpP* variants and Tn*6786*-related ICEs. The prevalence of all the 37 *crpP* variants (a) and of all the four groups of Tn*6786*-related ICEs (b) in the 200 *P. aeruginosa* isolates sequenced in this study are plotted

The draft genome sequences, i.e. assembled contigs, of the 200 clinical *P. aeruginosa* isolates from China were used for screening for the presence/absence of all the 37 *crpP* variants and all the four groups of Tn*6786*-related ICEs. There were 107 (53.5%) *crpP*-positive isolates, which included 97 carrying a sole *crpP* variant together with 10 harboring two *crpP* variants (Figure 4(a), and Supplementary material Table S1). This led to a total count of 117 *crpP* variants in 107 isolates. A total of nine distinct *crpP* variants, including *crpP-1.2* (39/117, 33.3%), *crpP-1.18* (21/117, 17.9%), *crpP-1.17* (18/117, 15.4%), *crpP-1.10* (16/117, 13.7%), *crpP-1.19* (15/117, 12.8%), *crpP-1.31* (3/117, 2.6%), *crpP-1.1* (2/117, 1.7%), *crpP-1.16* (2/117, 1.7%), and *crpP-1.20* (1/117, 0.9%), were identified in these 107 *crpP*-positive isolates. Each detected *crpP* gene was carried by a Tn*6786*-related ICE, and the 117 ICEs detected in these 107 isolates were composed of 67 group A ones, 34 group B ones, 11 group D ones, and 5 group C ones (Figure 4(b), and Supplementary material Table S1). Additionally, all these ICEs were integrated at the end of the tRNA^Lys^ gene as described in a previous report [14].

These 200 *P. aeruginosa* isolates could be assigned into 79 different STs, of which 43 and 44 corresponded to *crpP*-positive and -negative isolates, respectively (Supplementary material Table S1). Furthermore, the most prevalent STs for the 107 *crpP*-positive isolates were ST244 (16/107, 15.0%), ST235 (13/107, 12.1%), ST3418 (8/107, 7.5%), and ST277 (6/107, 5.6%), while the remaining 39 ones accounted for less than 60% of these 107 isolates. Among 43 STs identified from the *crpP*-positive isolates, ST244, ST235, ST274, and ST277 were recognized as the high-risk clones [22,23]. Additionally, a total of seven novel ST variants, namely ST3418 to ST3424, were identified in this study. In the present study, no correlation was found between MLST clones and ICEs/*crpP*.

### *Correlation between* crpP *genes and important resistance and virulence markers*

Among the 107 *crpP*-positive *P. aeruginosa* isolates, 71 (66.4%) were positive for other quinolone resistance markers, and 41 (38.3%) were positive for the virulence gene *exoU* [9,24]. Of the former 71 isolates, 63 (88.7%) possessed mutations in quinolone resistance determining regions (QRDRs), while 12 (16.9%) harbored *qnr* genes including *qnrVC1* (7/71, 9.9%), *qnrS2* (4/71, 5.6%), and *qnrVC6* (1/71, 1.4%). Statistical analysis was conducted to evaluate whether there was any correlation between *crpP* genes and these important markers (Supplementary material Table S8). Our results showed that compared to *crpP*-negative isolates, *crpP*-positive isolates harbored more *exoU* genes (*P* < 0.05).

### Conjugation experiments

To validate the intercellular mobility of *crpP*-carrying Tn*6786*-related ICEs, two ICEs harboring putative antibiotic resistance genes in addition to *crpP* (namely *emrAB*-carrying Tn*6783*, and *ampC*-carrying Tn*6786*) were chosen in this study for conjugal transfer experiments because the *crpP* genes were unsuitable as the selection markers based on antibiotic resistance. Yet, repeated conjugation attempts failed to transfer Tn*6783* or Tn*6786* from its wild-type isolate into ATCC 15,692.

### Summary of newly identified/designated MGEs

This study presented 17 newly identified MGEs, including two insertion sequence (IS) elements IS*Pa122* and IS*Pa127*, one integrative and mobilizable element (IME) Tn*6855b*, and 14 ICEs Tn*6783*, Tn*6784*, Tn*6785*, Tn*6786*, Tn*6789*, Tn*6790*, Tn*6791*, Tn*6794*, Tn*6795*, Tn*6796*, Tn*6797*, Tn*6826*, Tn*6827* and Tn*6829*. Additional 19 MGEs, i.e. seven IS elements IS*Pa120*, IS*Pa121*, IS*Pa123*, IS*Pa124*, IS*Pa125*, IS*Pa126* and IS*Pa128*, four unit transposons Tn*4661b*, Tn*6603b*, Tn*6811* and Tn*6812*, and eight ICEs Tn*6781*, Tn*6782*, Tn*6787*, Tn*6788*, Tn*6792*, Tn*6793*, Tn*6825* and Tn*6828* were newly designated in this study, but with previously determined sequences.

## Discussion

Since the *crpP-1.1* gene was initially identified in Mexico in 2018 [2], a total of 37 non-redundant *crpP* variants have been reported in at least 16 countries across North America, Asia, Africa, Oceania, and Europe [4–10]. However, conflicting names of *crpP* variants were presented in different reports. To solve this problem, herein we established a systematic typing/nomenclature scheme for all the available 37 CrpP variants based on their amino acid sequences. These 37 CrpP variants were divided into six primary groups, i.e. CrpP-1 to CrpP-6, where CrpP-1 comprised 32 variants, and each of CrpP-2 to CrpP-6 contained only a sole variant. Compared with CrpP-1.1, each of the other 31 CrpP-1 variants exhibited no more than 6 amino acid substitutions in total, indicating the variants within the primary group CrpP-1 were highly genetically conserved. Compared with CrpP-1.1, each of CrpP-2.1 to CrpP-6.1 displayed at least 39 amino acid substitutions in total, which indicated a much higher level of genetic diversity among different CrpP primary groups.

Among these 37 CrpP variants, 10 have been previously reported to mediate a decreased susceptibility to one or two quinolones, including CrpP-1.1/2.1/3.1/4.1/5.1/6.1 to ciprofloxacin [2,10], and CrpP-1.2/1.17/1.18/1.19 to ciprofloxacin and levofloxacin [5]. In this study, CrpP-1.1/2.1/3.1/4.1/5.1/6.1 as the representatives of the six primary CrpP groups were verified to mediate a decreased susceptibility to only ciprofloxacin, while CrpP-1.10/1.16/1.20/1.31 were disclosed, for the first time, to mediate a decreased susceptibility to both ciprofloxacin and levofloxacin. It was speculated that the Gly7Asp or Gly7HiS substitution, as observed in the eight CrpP-1 variants CrpP-1.2/1.10/1.16/1.17/1.18/1.19/1.20/1.31 relative to CrpP-1.1, might be the cause of a decreased susceptibility to levofloxacin besides ciprofloxacin, regardless of the occurrence of other amino acid substitutions [5].

Although *crpP* was originally identified in plasmid pUM505 [2], ICEs were identified as a major reservoir of *crpP* genes in *P. aeruginosa* [14]. Our results revealed that all the fully sequenced *crpP*-carrying ICEs had the highly conserved 45-bp terminal *attL*/*attR* pairs and displayed similar backbone gene organizations, and thus were assigned into a newly identified Tn*6786* family. A phylogenetic analysis based on their *int* sequences or on their core backbone SNPs could divide all the fully sequenced Tn*6786*-realted ICEs into four separate clustering groups (data not shown) that were designated as groups A to D. This showed that *crpP*-carrying Tn*6786*-related ICEs exhibited high-level diversity in nucleotide sequences.

A further detailed genetic dissection analysis was applied on the representative 22 *crpP*-carrying Tn*6786*-related ICEs, which covered all the above A to D groups. This analysis identified at least 97 events of exogenous gene acquisition, 69 events of deletion of backbone regions, and 10 events substitutions of backbone regions, which occurred at 13 regions/sites across the whole genomes of these 22 ICEs and most likely resulted from complex transposition and homologous recombination. In particular, massive gene acquisition/loss occurred across the whole genomes of Tn*6786*-related ICEs. Taken together, these 22 ICEs revealed a highly mosaic modular structures.

Acquisitions of different kinds of beneficial genes in addition of *crpP* were identified in this work: the inserted region*_orf228_* from Tn*6786* harbored the putative β-lactam resistance gene *ampC* [25]; the inserted region*_dsbG_* from Tn*6783* contained two putative multidrug resistance efflux pump genes *emrAB* [26]; Tn*6488* [27] from three ICEs harbored mercuric resistance *mer* locus; the inserted region*_orf1482_* from Tn*6781* harbored the *cup* locus encoding bacterial adhesive organelles [28]; the inserted region*_orf549_* from Tn*6781* and the inserted region*_pilL_* from other three ICE harbored *pyoS5* that mediated bactericidal activity against other *P. aeruginosa* isolates through encoding Pyocin S5 [29]. Acquisitions of exogenous genes involving drug resistance, virulence, and intra-species competition would enhance the adaptation to different niches of host *P. aeruginosa* strains.

To the best of our knowledge, this is the first study that reported on the characterization of *crpP* genes and *crpP*-carrying ICEs in China. 53.5% of the clinical *P. aeruginosa* isolates from China tested carried the *crpP* genes, which were exclusively located within chromosome-borne Tn*6786*-related ICEs. The herein detected *crpP*-positive rates in Chinese *P. aeruginosa* isolates were comparable to European isolates (46%) [5] and Australian isolates (43%) [9]. The *crpP*-carrying *P. aeruginosa* isolates were identified from all the 18 hospitals in the 12 Chinese cities studied, indicating wide distribution of these isolates in the hospital settings in China. Notably, thirty-seven (34.6%) of the 107 *crpP*-positive *P. aeruginosa* isolates from China belonged to the high-risk clones ST244, ST235, ST274 and ST277, with ST235 being worldwide distributed [22,23].

Considering the high-level diversification in both nucleotide sequences and modular structures, the *crpP*-carrying Tn*6786*-related ICEs were at active stages of evolution and showed the high potential to act as an important vector for dissemination of antibiotic resistance genes besides *crpP*.

Although a circular form of *crpP*-carrying ICEs has been previously confirmed through PCR assays [5], repeated conjugation attempts failed to transfer Tn*6783* or Tn*6786* from their wild-type isolate into ATCC 15,692, whose chromosome (accession number NC_002516) did have the conserved tRNA^Lys^ gene. This failure might result from the lesions in the conjugal transfer regions of these two tested ICEs.

The type III secretion system effector cytotoxin ExoU could markedly enhance the virulence of *P. aeruginosa* [24]. The *exoU* gene was also considered to be associated with fluoroquinolone-resistant strains of *P. aeruginosa* [9,30,31]. Our results further proved that the *exoU* gene was positively correlated with *crpP* genes. The mechanisms underlying the co-carriage of these two genes in *P. aeruginosa* strains need to be further studied.

In conclusion, systematic typing/nomenclature schemes for *crpP* variants and *crpP*-carrying Tn*6786*-related ICEs were established. Ten major representative CrpP enzymes were confirmed to mediate a limited decreased susceptibility to quinolones. 53.5% of the tested clinical *P. aeruginosa* isolates from China carried the *crpP* genes, which were exclusively located within chromosome-borne Tn*6786*-related ICEs. Tn*6786*-related ICEs displayed high-level diversification in both nucleotide sequences and modular structures. Especially, massive gene acquisition/loss occurred across the whole genomes of Tn*6786*-related ICEs. This work provided a deeper insight into the bioinformatics and epidemiology of *crpP* genes and *crpP*-carrying Tn*6786*-related ICEs.

## Supplementary Material

Supplemental Material

## Data Availability

The data used to support the findings of this study are available from the corresponding author upon request (dongshengzhou1977@gmail.com).
